# The effect of resistance inspiratory muscle training in the management of breathlessness in patients with thoracic malignancies: a randomised controlled trial

**DOI:** 10.1007/s00520-025-09511-9

**Published:** 2025-05-22

**Authors:** Meng-Yuan Li, Xian-Liang Liu, Bo Peng, Tao Wang, Li-Qun Yao, Hou-Qiang Huang, Wai Hang Kwok, Jing-Yu Benjamin Tan, Alex Molassiotis

**Affiliations:** 1 School of Nursing and Midwifery, University of Southern Queensland, Ipswich, QLD Australia; 2 School of Nursing and Health Sciences, Hong Kong Metropolitan University, Kowloon, Hong Kong SAR China; 3 Faculty of Health, Charles Darwin University, Brisbane Centre, Brisbane, QLD Australia; 4 School of Nursing, The Hong Kong Polytechnic University, Kowloon, Hong Kong SAR China; 5 Affiliated Hospital of Southwest Medical University, Luzhou, Sichuan China; 6 Centre for Health Research, University of Southern Queensland, Springfield, QLD Australia; 7 College of Nursing, Fujian University of Traditional Chinese Medicine, Fuzhou, Fujian China; 8 School of Nursing and Midwifery, Edith Cowan University, Joondalup, WA Australia; 9 Faculty of Health, Charles Darwin University, Darwin, NT Australia; 10 College of Arts, Humanities & Education, University of Derby, Derby, UK

**Keywords:** Lung cancer, Inspiratory muscle training, Breathlessness, Quality of life, Randomised controlled trial

## Abstract

**Purpose:**

To assess the effects of resistance inspiratory muscle training (IMT) on breathlessness in patients with thoracic malignancies.

**Methods:**

This is a two-arm, non-blinded, randomised controlled trial (RCT). A total of 196 participants were randomly assigned (1:1) into two groups: a control group (routine care) and an intervention group (routine care + IMT training using a pressure threshold device). The intervention duration was 12 weeks with 30 min/day, 5 days/week. The primary outcome was breathlessness severity, assessed by the modified Borg scale (mBorg). Secondary outcomes were worst and average breathlessness over the past 24 h (assessed by the 11-point Numerical Rating Scale), breathlessness severity (assessed by the Dyspnoea-12, D-12), the 6-min walk distance (assessed by the 6-min walk test, 6MWT), quality of life (assessed by the St George’s Respiratory Questionnaire, SGRQ), and emotional status (assessed by the Hospital Anxiety and Depression Scale). Assessments were conducted at baseline (T1), week 8 (T2), and week 12 (T3). Adjusted generalized estimating equations (GEE) models for repeated measures over time were performed using the Statistical Package for Social Science (SPSS) software. The modified intention-to-treat principle was used for data analysis.

**Results:**

Of the 196 participants, 190 completed the trial, and six dropped out. 31.63% of participants completely adhered to the required sessions of IMT. In the adjusted GEE model, statistical and minimal clinically important differences were observed on the m-Borg score at week 8 (*P* = 0.002), while no significant group-by-time effect was observed in the mBorg. Compared with the control group and baseline, participants in the intervention group showed a significant reduction in D-12 total scores at week 8 (*P* = 0.005) and week 12 (*P* = 0.004). No significant group-by-time interaction effects were observed for worst and average breathlessness over the past 24 h, anxiety, depression, 6MWT, and SGRQ scores.

**Conclusions:**

This study highlights the short-term benefits of IMT for reducing breathlessness among patients with thoracic malignancies. However, the long-term effects should be explained with caution due to the participants’ suboptimal adherence. Future studies should explore different strategies to improve adherence and further evaluate the sustained effects of IMT over time.

**Trial registration.:**

ClinicalTrials.gov NCT03834116.

**Date of registration.:**

2019–02-06.

## Introduction

Thoracic malignancies have become a significant global health burden with high morbidity and mortality, particularly lung cancer (LC), which is the leading cause of cancer mortality worldwide [1]. In China, patients diagnosed with LC ranked first, with new cases of 815,000 and 714,000 deaths in 2020 [2]. Due to the cancer progression and side effects of cancer treatments, LC patients commonly experience breathlessness [3]. Reductions in expiratory and inspiratory muscle strength can persist up to 12 weeks following thoracotomy in LC [4]. Research has reported that the weighted grand mean prevalence of breathlessness in LC patients was 34.9% [5], which has a detrimental impact on overall well-being, and impedes patients from engaging in daily activities [6]. Management of breathlessness in LC patients is generally focusing on medication and oxygen as prescribed [7]. However, breathlessness tends to be more refractory to treatment than other symptoms (e.g., pain) and less responsive to medications, making it a poorly controlled symptom [8]. Considering the uncertainty of breathlessness occurrence, oxygen therapy may not always be readily accessible and self-administered when needed. Additionally, the utilisation of home oxygen therapy can be limited due to its high costs and side effects (e.g., risk of epistaxis related to the irritation of nasal cannula) [9].

Inspiratory muscle training (IMT) refers to a targeted strengthening of the inspiratory muscles by applying resistance during inspiration [10]. It has been used for respiration symptom relief since the 1980 s [10]. The most common method of providing IMT is inspiratory threshold pressure loading [11], which has demonstrated beneficial effects on quality of life (QoL), exercise capacity, and breathlessness in non-cancer respiratory diseases over a long time [12]. IMT requires participants to breathe against set resistance, which increases the workload of the breathing muscles. With continued practice and progressive resistance, IMT improves the strength and endurance of the inspiratory muscles [13]. Enhancement in inspiratory muscle strength and endurance could be in increasing the inspiratory flow, decreasing the inspiratory time, and improving the expiratory time, consequently reducing the sensation of breathlessness during daily activities [11]. In a clinical trial conducted by Liu et al. [13], the recovery of respiratory muscle strength was well documented in LC patients after a combination of IMT and aerobic exercise training. Considering the reduction of breathing muscles resulting from the long-term impact of cancer treatments, IMT could be a potentially encouraging intervention for managing breathlessness in patients with thoracic malignancies. Moreover, cancer and its treatment have numerous impacts that can be counteracted through IMT, potentially relieving breathlessness [14]. These include improvements in lung function, respiratory muscle sarcopenia, loss of chest mobility due to surgery, and pulmonary fibrosis caused by chest radiotherapy [15, 16].

A latest systematic review assessing the various breathing exercises on breathlessness and QoL in LC patients, highlighted the promising role of IMT in enhancing the well-being of these patients [17]. Another meta-analysis indicated that IMT is effective in improving pulmonary function in cancer patients, particularly highly recommending its application in LC patients [18]. However, IMT did not yield statistically significant results on exercise capacity and QoL due to the limited sample size and unsatisfactory methodology of the included studies [18]. Ambiguous results of IMT on exercise capacity, maximal inspiratory pressure, maximal expiratory pressure, and QoL were reported by one systematic review with only five trials on postoperative patients with LC [14]. In addition, strategies using the IMT for improving breathlessness management and QoL in LC patients have been reported in the literature [16, 19]. However, due to the limited sample size, and wide heterogeneity in the utilisation of IMT, full-scale and high-quality randomised controlled trials (RCTs) are needed to confirm the effects of IMT in patients with thoracic malignancies. To the best of our knowledge, no large-scale RCT has been conducted so far to assess IMT aiming to reduce breathlessness in thoracic malignancies. A pilot study in patients with clinically stable LC was conducted by Molassiotis et al. [16], showing that IMT was not only feasible, acceptable, and safe for LC patients, but also associated with statistical and clinical improvements in the breathlessness-related parameters, depression and QoL. Following the modification of the RCT study protocol, a fully powered RCT was conducted to draw definitive conclusions on the effects of IMT on breathlessness among patients with thoracic malignancies.

## Methods

### Design and participants

The study methods followed the previous pilot study [16] and the CONSORT guideline. A two-arm, non-blinded, RCT was utilised. Recruitment took place in the Affiliated Hospital of Southwest Medical University (Sichuan, Mainland China).

Adult participants were eligible for recruitment if they (1) were diagnosed with primary LC or mesothelioma (histological diagnosis); (2) have an expected prognosis (over 3 months) as determined by the clinicians; (3) have refractory breathlessness and have not responded to current treatment for the past two weeks as determined by the clinicians; and (4) have an oxygen saturation above 85% at rest. Participants were ineligible if they (1) were with unstable COPD and the condition was under acute or frequent exacerbation; (2) were rapidly worsening breathlessness and needed urgent medical intervention; (3) have palliative radiotherapy to the chest received within four weeks or chemotherapy within two weeks; and (4) were experiencing intractable cough and have unstable angina or clinically significant pleural effusion requiring drainage.

The estimation of sample size was based on the primary outcome of the modified Borg scale (mBorg) score. The change score of mBorg from baseline to 3-month assessment was 0.80 and the established minimally important difference was 1 according to the pilot study [16]. Since a 25% attrition was reported in the pilot study [16], the required sample size was 196 totally (98 in each group).

### Randomisation and blinding

Two arms (a control arm and an IMT arm) were designed. An independent statistician prepared the computer-generated random sequence. After completion of the baseline assessment of an eligible participant, the investigator (responsible for recruitment) contacted the independent statistician to request the group allocation. Participants included in the study were told what was being applied. The blinding design of the participants, investigators, and outcome assessors was not achieved given that most of the outcome measures were self-reported and the participants were deemed as the outcome assessors. A single blinding design (outcomes assessor) was described in the registered clinical trial protocol, and this paper acknowledges this minor discrepancy.

### Study interventions

All the participants received routine methods of care (‘standard treatment’ as indicated in the registered clinical trial protocol, which was utilised in both groups as usual care), including standard health education (pain management, medication management, exercise guidance, lifestyle adjustment) and regular follow-ups. Participants in the intervention group received additional IMT intervention. The IMT intervention was detailed in the published pilot study [16]. Participants in the IMT group used a threshold inspiratory muscle training device from Phillips Respironics for five days per week, 30 min per day, for 12 weeks. The daily 30-min training can be divided into two sessions. Before initiating the intervention, participants’ maximum inspiratory pressure (MIP) was measured by using an inspiratory pressure measuring instrument as a baseline. Then participants were required to perform 3–5 min of exercise under the Research Assistant’s supervision to assess whether they had encountered difficulties (e.g., tiredness or shortness of breath) in completing the entire session. The IMT exercise intensity was determined based on the measured participants’ MIP. Specifically, the initial training intensity started from 40% MIP, with a weekly increase of 5% until reaching 70% MIP as the maximum intensity [16]. To maintain the effectiveness of IMT usage, a nose clip was suggested to prevent patients from inhalation through the nose to overcome the resistance from the valve. If using a nose clip is uncomfortable for the participants, the participants were suggested to manually pinch the nostrils with their hands during the IMT exercise. The inspiratory duration lasted for 1.5 to 2 s, while the expiratory duration was extended to 6 s, maintaining an inhalation-to-exhalation (IE) ratio of 1:3 [20]. This aims to enhance tidal volume, reduce dead space ventilation, improve alveolar ventilation, decreases respiratory effort, and alleviates breathlessness symptoms [20]. Consequently, the respiratory rate was maintained at about 8 breaths per minute.

### Study procedures

Participants’ recruitment was conducted at the outpatient clinic of study site. Eligible participants who provided written informed consent were recruited. Baseline assessments were performed before randomisation. Participants allocated to the intervention group received IMT training using a pressure threshold device. A study investigator explained and demonstrated how to use and adjust the exercise intensity until the participants mastered the skills with a return demonstration. During the intervention period, participants in the IMT group received weekly reminders to increase the IMT’s resistance level and concurrently monitor their exercise progress. Participants in the intervention group were also required to document their IMT practice sessions. Instruction videos on the use of the pressure threshold device were provided to the participants in the IMT group, ensuring that they could strengthen the IMT technique at home. Consistency in each stage of this study was maintained by a detailed protocol, constant supervision of the research activities, and regular meetings among the study investigators.

Demographic and clinical characteristics of patients were collected face-to-face at baseline (T1). All the clinical outcomes were assessed via either face-to-face (for participants who returned to the study site) or telephone (for those who were unable to return due to the COVID policies) immediately after the completion of the intervention (8-week, T2) and the completion of the follow-up (12-week, T3). Since all the subjective outcomes were self-administrated questionnaires, the participants were encouraged to complete the questionnaires independently. Only necessary interpretation was provided in a neutral way when the participants had confusions about terms or items in the questionnaires.

### Outcome measures

#### Baseline assessment

The participants’ sociodemographic data, medical history, medication regimen, and other baseline data were collected via a predesigned baseline data collection form. The spirometry assessment at baseline was also recorded.

#### Primary outcome

##### Severity of breathlessness: modified Borg scale (mBorg)

The mBorg is a vertical 11-point scale, from ‘0’ (nothing at all) to ‘10’ (maximal) to assess the level of breathlessness at the time of assessment. A higher score represents a higher breathlessness level [21]. The within-groups minimal clinically important difference (MCID) of 1 point in the mBorg score was deemed to indicate a clinically significant change in breathlessness [21].

#### Secondary outcomes

##### Severity of breathlessness: Dyspnea-12 Questionnaire (D-12)

The D-12 obtained an overview of the severity of breathlessness, consisting of both physical aspect and affective aspect [22]. Each item was rated from ‘0’ (none) to ‘3’ (severe), with a total score range of 0 to 36. A higher score reflects a greater severity of breathlessness [22]. The D-12 has been widely used for the assessment of breathlessness in patients with lung disease [22]. The Chinese version of D-12 exhibited strong internal consistency, with Cronbach’s alpha coefficients of 0.83 in lung cancer patients [23].

##### Severity of breathlessness over ‘the past 24 h’: Numerical Rating Scale (NRS)

This study also used a 0–10 NRS (0 = no,10 = as bad as can be) to assess the ‘average’ level and ‘worst’ level of the severity of breathlessness and the distress caused by breathlessness over ‘the past 24 h’ [24]. The NRS has been validated and used for the severity of breathlessness [24].

##### Exercise capacity: the 6-min walk test (6MWT)

The exercise capacity was assessed using the 6-min walk test (6MWT), which is patient-friendly, well-validated, and exhibits strong correlations with lung function in clinical trials [25]. In accordance with the American Thoracic Society (ATS) guidelines, participants were directed to a quick walk back and forth and stop or rest as necessary on a demarcated 30-m linear path on a flat surface. At each minute, reminders of the time remaining were provided to participants to ensure consistency with standardized procedures [25]. Following the completion of 6 min, the test administrator recorded the 6-min walk distance (6MWD). During the baseline assessment, the 6MWT was conducted in person by the researcher who provided detailed instructions explaining the procedure and safety precautions. As the 6MWT is simple to administer, requiring no specialized equipment or advanced training [26], participants were therefore provided standardized instructions on how to self-administer the 6MWT at home using their phones during the COVID-19 outbreak. The instructions were as follows: (1) participants were instructed to wear comfortable clothing and supportive shoes; (2) choose a flat, straight walking path recorded in length; (3) a smartphone timer was set for a 6-min countdown; (4) the timer was started when the participant began walking, and they stopped immediately when the timer rang; (5) distance was recorded by measuring the number of laps completed along their designated walking path; and (6) participants were advised to have a family member or caregiver present during the test in case of any issues.

##### Health-related QoL: St George’s Respiratory Questionnaire (SGRQ)

The SGRQ was specifically designed to measure and quantify health-related status in patients with chronic airflow limitation, and it has been validated for assessing QoL in patients with lung disease (including lung cancer patients) [27, 28]. The SGRQ consists of 50 items with 76 weighted responses, yielding into ‘symptoms’, and ‘activity’, ‘impact’ sections [29]. The total score also summarizes the impact of the disease on overall health status ranging from 0–100, where a lower score indicates better health [29].

##### Emotional Status: Hospital Anxiety and Depression Scale (HADS)

The HADS is a 14-ite scale for measuring anxiety and depression. It has been validated in cancer patients [30]. The items are rated on a 4-point scale from 0 (not present) to 3 (considerable), with higher scores representing more anxiety/depression. The Mandarin Chinese version has a satisfactory psychometric property, with Cronbach’s alpha ≥ 0.840 in the total and subscales [30].

### Data analysis

Data was analyzed using the IBM SPSS 25.0 software. The statistical significance was set at a *p-*value of < 0.05. The participants’ demographic and clinical characteristics were reported as frequencies and percentages or means and standard deviations. Categorical variables were analyzed using the chi-square test or independent t-test to conduct baseline comparisons. The description of normally and non-normally distributed continuous data was reported as mean (standard deviation [SD]) and median (interquartile range [IQR]), respectively. Mann–Whitney U test was conducted to compare non-parametric variables while independent *t*-test was used for the comparison of normally distributed variables. Generalized estimating equations (GEE) models were used to compare differences in the outcomes (mBorg, NRS, D-12, 6MWT, SGRQ, HADS) between the two groups across multiple time points (T1-T2-T3) with adjustment for potential confounding factors. The potential confounding factors are variables that were significantly different between the two groups at baseline (BMI, D-12 score) and other potential covariates (age, weight, smoking, exercise, insomnia) [31, 32]. The GEE model accounts for the following: (1) group effect: assumes the outcome varies based on group assignment, independent of time; (2) time effect: assumes the outcome varies over time, independent of group assignment; and (3) group-by-time effect (main effect): examines the interaction between group assignment and time, comparing the intervention group at later time points with both the baseline and the control group at all time points [33]. The group-by-time effect is the primary result in this analysis, indicating the divergence in outcome trajectories over time between groups. A modified intention-to-treat analysis was followed.

## Results

### Sociodemographic and clinical characteristics

**Fig. 1:**
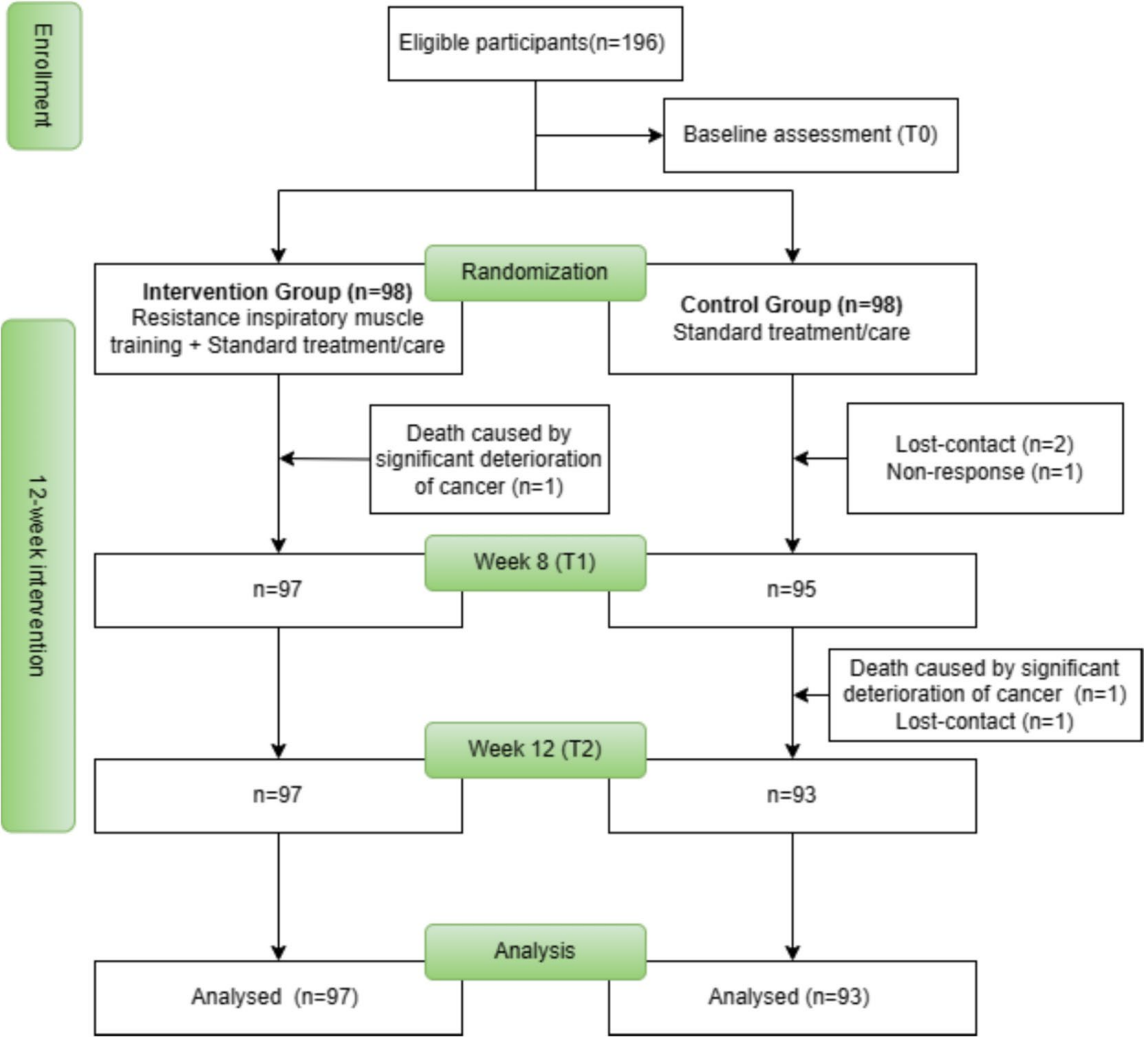
CONSORT diagram for the study population

A total of 196 participants were recruited and randomly allocated to each group (Fig. 1). The attrition rate was 3%. Table 1 shows the baseline characteristics of the participants. No statistically significant between-group differences were found in baseline characteristics except the total score of D-12 (*P* = 0.02) and BMI (*P* = 0.02) which were introduced as two of the potential confounders in the adjusted GEE model. In the IMT group, approximately one-third of participants (31.63%) adhered strictly to the IMT protocol. The remaining participants (68.37%) completed most of the required training sessions due to malfunctioning IMT devices and discontinued monitoring strategies resulting from the implementation of COVID-19 policies.

**Table 1:** Baseline demographic and clinical characteristics of the participants

Variables		Control group	Intervention group	Value	*P*
		*n* (%)	*n* (%)		
Educational background (*n* = 196, 98/98)	No formal education	9 (9.2)	11 (11.2)	2.28^A^	0.81
	Primary school	26 (26.5)	24 (24.5)		
	Secondary school	33 (33.7)	38 (38.8)		
	High school/technical school	12 (12.2)	9 (9.2)		
	Junior college	8 (8.2)	10 (10.2)		
	Bachelor’s degree or above	10 (10.2)	6 (6.1)		
Gender (*n* = 196,98/98)	Male	50(51.0)	41(41.8)	1.66^A^	0.20
	Female	48(49.0)	57(58.2)		
Marital status (*n* = 196, 98/98)	Single	1 (1.0)	1 (1.0)	0.00^A^	1.00
	Married	97 (99.0)	97 (99.0)		
Occupation (*n* = 194, 98/96)	Professional and technical personnel	5(5.1)	11 (11.5)	4.30^A^	0.37
	Labour worker	29 (29.6)	20 (20.8)		
	Clerical or administrative worker	4 (4.1)	6 (6.3)		
	No longer working*	39 (39.8)	38 (39.6)		
	Other	21 (21.4)	21 (21.9)		
Household income (RMB) (*n* = 178, 90/88)	< 3000	54 (60.0)	52 (59.1)	1.43^A^	0.70
	3000–6000	30 (33.3)	31 (35.2)		
	> 6000–10000	6 (6.7)	4 (4.5)		
	> 10,000	0 (0)	1 (1.1)		
Source of healthcare insurance (*n* = 195, 98/97)	New Rural Cooperative Medical System	42 (42.9)	46 (47.4)	0.86^A^	0.65
	Resident Medical Insurance/Employee Medical Insurance	55 (56.1)	49 (50.5)		
	Self-paid	1 (1.0)	2 (2.1)		
BMI (*n* = 194,98/98)	Underweight (< 18.5)	9 (9.2)	3 (3.1)	5.64^A^	0.13
	Normal/healthy weight (18.5 ~ 22.9)	43 (43.9)	42(42.9)		
	Overweight (23 ~ 24.9)	25 (25.5)	21(21.4)		
	Obese (≥ 25)	21 (21.4)	32 (32.7)		
Cancer stage (*n* = 183, 94/89)	I	63 (67.0)	61 (68.5)	5.63^A^	0.47
	IIA	4 (4.3)	4 (4.5)		
	IIB	7 (7.4)	3 (3.4)		
	IIIA	6 (6.4)	9 (10.1)		
	IIIB	6 (6.4)	2 (2.2)		
	IIIC	1 (1.1)	0 (0)		
	IV	7 (7.4)	10 (11.2)		
Surgery types (*n* = 169,87/82)	Left upper lobectomy	20 (23.0)	26 (31.7)	3.04^A^	0.69
	Left lower lobectomy	6 (6.9)	6 (7.3)		
	Right upper lobectomy	28 (32.2)	23 (28.0)		
	Right lower lobectomy	19 (21.8)	14(17.1)		
	Microwave ablation	0 (0)	1 (1.2)		
	Other	14 (16.1)	12 (14.6)		
Hypertension (*n* = 195, 98/97)	Yes	19 (19.4)	26 (26.8)	1.51^A^	0.22
	No	79 (80.6)	71 (73.2)		
Diabetes (*n* = 196,98/98)	Yes	4 (4.1)	11 (11.2)	2.60^A^	0.11
	No	94 (95.9)	87 (88.8)		
Asthma (*n* = 196,98/98)	Yes	2 (2.0)	2 (2.0)	0.00^A^	1.00
	No	96 (98.0)	96 (98.0)		
Pneumonectasis (*n* = 196,98/98)	Yes	3 (3.1)	5 (5.1)	0.13^A^	0.72
	No	95 (96.9)	93 (94.9)		
Pulmonary tuberculosis (*n* = 196,98/98)	Yes	3 (3.1)	3 (3.1)	0.00^A^	1.00
	No	95 (96.9)	95 (96.9)		
Heart diseases (*n* = 194,96/98)	Yes	7 (7.3)	5 (5.1)	0.40^A^	0.53
	No	89 (92.7)	93 (94.9)		
Targeted therapy (*n* = 196,98/98)	Yes	23(23.5)	23(23.5)	0.00^A^	1.00
	No	75(76.5)	75(76.5)		
Exercise (Hour/week) (*n* = 195, 98/97)	0–2	79 (80.6)	78 (80.4)	1.36^A^	0.72
	3–4	11 (11.2)	14 (14.4)		
	5–6	6 (6.1)	3 (3.1)		
	More than 6	2 (2.0)	2 (2.1)		
Smoking (*n* = 195, 98/97)	Never smoked	64 (65.3)	65 (67.0)	0.35^A^	0.84
	Current smoking	2 (2.0)	1 (1.0)		
	Previous smoking	32 (32.7)	31 (32.0)		
Alcohol consumption (*n* = 196, 98/98)	Yes	34 (34.7)	37 (37.8)	0.20^A^	0.66
	No	64 (65.3)	61 (62.2)		
Insomnia (*n* = 196, 98/98)	Yes	38 (38.8)	34 (34.7)	0.35^A^	0.55
	No	60 (61.2)	64 (65.3)		
		**Mean ± SD**	**Mean ± SD**		
Age (*n* = 196, 98/98)		58.8 ± 5.10	58.78 ± 9.75	0.05^B^	0.96
BMI index		22.64 ± 2.79	23.63 ± 3.27	− 2.28^B^	0.02
Blood oxygen level (%) (*n* = 196,98/98)		97.58 ± 1.47	97.63 ± 1.37	4870.50^C^	0.85
6MWD (*n* = 194, 96/98)		389.82 ± 60.90	393.04 ± 57.45	4830.00^C^	0.75
mBorg score (*n* = 195, 97/98)		4.25 ± 1.71	4.21 ± 1.36	4848.50^C^	0.80
SGRQ total score (*n* = 196, 98/98)		27.06 ± 12.72	29.29 ± 13.39	5294.50^C^	0.22
HADS-D score (*n* = 191, 94/97)		1.74 ± 2.51	1.92 ± 2.59	4653.00^C^	0.80
HADS-A score (*n* = 195, 97/98)		2.96 ± 2.42	3.19 ± 2.91	4746.00^C^	0.99
D-12 total score (*n* = 192, 95/97)		7.13 ± 3.78	8.16 ± 3.93	5537.50^C^	0.02
NRS-worst score (*n* = 195, 97/98)		4.36 ± 2.23	4.53 ± 1.94	4979.00^C^	0.56
NRS-average score (*n* = 195, 97/98)		2.54 ± 1.35	2.54 ± 1.26	4805.00^C^	0.89

^A^, Chi square test; ^B^, independent *t*-test; ^C^, Mann–Whitney *U* test; *, including housewife, unemployment, and retired; *6MWD*, 6-min walk distance; *mBorg*, modified Borg scale; *SGRQ*, St George’s Respiratory Questionnaire; *HADS*, Hospital Anxiety and Depression Scale; *D-12*, Dyspnea-12 Questionnaire; *NRS*-*worst*, worst breathlessness over the past 24 h assessed by the Numerical Rating Scale; *NRS-average*, average breathlessness over the past 24 h assessed by the Numerical Rating Scale

### Primary outcome

#### mBorg

Significant within-group differences were found in both the intervention group and control group, with its mBorg score at T2 and T3 being significantly lower than the score at baseline (all *P* < 0.05, Table 2), suggesting the breathlessness was reduced over time. The observed change in mBorg score in the IMT group at both T2 and T3 reached the minimally important clinical difference (MCID) of 1, while the MCID of mBorg score in the control group was observed until at T3. Group-comparison showed that the mBorg score of the control group was significantly higher (worse breathlessness) than the IMT group at T2 (*P* = 0.002). The group-by-time effect was not significant for the mBorg score (Table 3).

**Table 2:** Group-effect and time-effect of intervention on outcomes by adjusted GEE model

Outcome	Timepoint	Comparison	Adjusted				
			MD	SE	EF	${\chi }^{2}$	*P*
mBorg	T1	IG VS. CG	− 0.300	0.19	0.22	2.46	0.117
	T2	IG VS. CG	− 0.515	0.16	0.45	9.79	**0.002**
	T3	IG VS. CG	− 0.240	0.16	0.21	2.18	0.140
	IG	T3 VS. T2	− 0.125	0.08	0.23	2.51	0.113
		T3 VS. T1	− 0.663	0.13	0.72	24.83	** < 0.001**
		T2 VS. T1	− 0.538	0.10	0.76	28.20	** < 0.001**
	CG	T3 VS. T2	− 0.400	0.07	0.78	28.79	** < 0.001**
		T3 VS. T1	− 0.723	0.12	0.85	34.10	** < 0.001**
		T2 VS. T1	− 0.324	0.13	0.37	6.52	**0.011**
D-12-total	T1	IG VS. CG	0.967	0.46	0.30	4.34	0.037
	T2	IG VS. CG	− 0.304	0.42	0.11	0.53	0.468
	T3	IG VS. CG	− 0.440	0.42	0.15	1.10	0.294
	IG	T3 VS. T2	− 0.434	0.13	0.48	11.13	**0.001**
		T3 VS. T1	− 2.743	0.32	1.24	74.09	** < 0.001**
		T2 VS. T1	− 2.309	0.31	1.06	54.17	** < 0.001**
	CG	T3 VS. T2	− 0.298	0.17	0.26	3.18	0.075
		T3 VS. T1	− 1.336	0.41	0.48	10.62	**0.001**
		T2 VS. T1	− 1.038	0.36	0.42	8.32	**0.004**
D-12-physical	T1	IG VS. CG	0.716	0.37	0.28	3.73	0.053
	T2	IG VS. CG	− 0.302	0.37	0.12	0.66	0.415
	T3	IG VS. CG	− 0.416	0.36	0.17	1.31	0.253
	IG	T3 VS. T2	− 0.499	0.12	0.60	17.36	** < 0.001**
		T3 VS. T1	− 2.406	0.26	1.34	87.99	** < 0.001**
		T2 VS. T1	− 1.907	0.23	1.17	66.31	** < 0.001**
	CG	T3 VS. T2	− 0.385	0.14	0.40	7.45	**0.006**
		T3 VS. T1	− 1.275	0.31	0.61	17.38	** < 0.001**
		T2 VS. T1	− 0.890	0.26	0.49	11.46	**0.001**
D-12-emotionnal	T1	IG VS. CG	0.267	0.19	0.20	1.97	0.160
	T2	IG VS. CG	− 0.003	0.11	< 0.01	< 0.01	0.977
	T3	IG VS. CG	− 0.039	0.12	0.05	0.11	0.740
	IG	T3 VS. T2	0.051	0.04	0.20	1.93	0.164
		T3 VS. T1	− 0.364	0.15	0.35	5.95	**0.015**
		T2 VS. T1	− 0.415	0.15	0.41	7.96	**0.005**
	CG	T3 VS. T2	0.086	0.05	0.25	3.03	0.082
		T3 VS. T1	− 0.059	0.16	0.05	0.13	0.714
		T2 VS. T1	− 0.145	0.15	0.14	0.90	0.344
NRS-worst	T1	IG VS. CG	0.034	0.27	0.02	0.02	0.902
	T2	IG VS. CG	− 0.247	0.20	0.18	1.53	0.217
	T3	IG VS. CG	0.101	0.19	0.08	0.29	0.588
	IG	T3 VS. T2	− 0.014	0.08	0.03	0.03	0.859
		T3 VS. T1	− 0.390	0.17	0.33	5.26	**0.020**
		T2 VS. T1	− 0.380	0.15	0.37	6.77	**0.009**
	CG	T3 VS. T2	− 0.360	0.09	0.56	14.69	** < 0.001**
		T3 VS. T1	− 0.460	0.16	0.41	8.06	**0.004**
		T2 VS. T1	− 0.100	0.15	0.10	0.44	0.507
NRS-average	T1	IG VS. CG	− 0.153	0.17	0.13	0.79	0.373
	T2	IG VS. CG	− 0.057	0.13	0.06	0.18	0.674
	T3	IG VS. CG	0.094	0.12	0.12	0.65	0.421
	IG	T3 VS. T2	− 0.126	0.07	0.27	3.60	0.058
		T3 VS. T1	− 0.166	0.14	0.17	1.45	0.228
		T2 VS. T1	− 0.041	0.12	0.05	0.12	0.734
	CG	T3 VS. T2	− 0.280	0.07	0.60	16.65	** < 0.001**
		T3 VS. T1	− 0.410	0.12	0.51	12.23	** < 0.001**
		T2 VS. T1	− 0.137	0.13	0.15	1.14	0.285
6MWD	T1	IG VS. CG	9.580	7.57	0.18	1.60	0.206
	T2	IG VS. CG	10.970	7.17	0.23	2.34	0.126
	T3	IG VS. CG	8.218	7.90	0.16	1.08	0.298
	IG	T3 VS. T2	7.750	4.21	0.28	3.40	0.065
		T3 VS. T1	18.457	6.43	0.42	8.23	**0.004**
		T2 VS. T1	10.707	4.81	0.32	4.96	**0.026**
	CG	T3 VS. T2	10.502	3.35	0.47	9.81	**0.002**
		T3 VS. T1	19.819	5.53	0.53	12.86	** < 0.001**
		T2 VS. T1	9.317	4.54	0.30	4.20	**0.040**
HADS-A	T1	IG VS. CG	− 0.077	0.37	0.03	0.04	0.836
	T2	IG VS. CG	0.079	0.32	0.04	0.06	0.807
	T3	IG VS. CG	0.088	0.31	0.04	0.08	0.775
	IG	T3 VS. T2	− 0.240	0.13	0.28	3.67	0.055
		T3 VS. T1	− 0.173	0.31	0.08	0.30	0.582
		T2 VS. T1	0.067	0.30	0.03	0.05	0.821
	CG	T3 VS. T2	− 0.249	0.13	0.28	3.65	0.056
		T3 VS. T1	− 0.338	0.27	0.18	1.55	0.213
		T2 VS. T1	− 0.088	0.26	0.05	0.11	0.736
HADS-D	T1	IG VS. CG	− 0.177	0.32	0.08	0.31	0.581
	T2	IG VS. CG	− 0.252	0.23	0.16	1.16	0.282
	T3	IG VS. CG	0.009	0.25	0.01	< 0.01	0.971
	IG	T3 VS. T2	0.093	0.11	0.12	0.76	0.385
		T3 VS. T1	− 0.163	0.29	0.08	0.31	0.577
		T2 VS. T1	− 0.256	0.28	0.13	0.84	0.361
	CG	T3 VS. T2	− 0.168	0.11	0.22	2.21	0.137
		T3 VS. T1	− 0.349	0.30	0.17	1.37	0.241
		T2 VS. T1	− 0.181	0.28	0.09	0.41	0.524
SGRQ-total	T1	IG VS. CG	0.838	1.16	0.10	0.53	0.469
	T2	IG VS. CG	1.114	0.85	0.19	1.70	0.193
	T3	IG VS. CG	0.556	0.82	0.10	0.46	0.497
	IG	T3 VS. T2	0.254	0.43	0.09	0.36	0.550
		T3 VS. T1	− 0.281	1.01	0.04	0.08	0.781
		T2 VS. T1	− 0.536	0.86	0.09	0.39	0.532
	CG	T3 VS. T2	0.813	0.46	0.26	3.17	0.075
		T3 VS. T1	0.001	1.04	< 0.01	< 0.01	0.999
		T2 VS. T1	− 0.811	1.03	0.11	0.62	0.430
SGRQ-symptom	T1	IG VS. CG	1.097	1.74	0.09	0.40	0.527
	T2	IG VS. CG	3.055	1.69	0.26	3.27	0.070
	T3	IG VS. CG	1.415	1.61	0.13	0.77	0.381
	IG	T3 VS. T2	− 2.443	0.82	0.43	8.89	**0.003**
		T3 VS. T1	− 1.879	1.51	0.18	1.55	0.213
		T2 VS. T1	0.564	1.38	0.06	0.17	0.684
	CG	T3 VS. T2	− 0.803	0.69	0.17	1.37	0.243
		T3 VS. T1	− 2.197	1.52	0.21	2.08	0.149
		T2 VS. T1	− 1.394	1.47	0.14	0.90	0.342
SGRQ-activity	T1	IG VS. CG	1.569	1.67	0.13	0.89	0.346
	T2	IG VS. CG	0.442	1.06	0.06	0.17	0.676
	T3	IG VS. CG	0.180	1.02	0.03	0.03	0.859
	IG	T3 VS. T2	0.027	0.56	0.01	< 0.01	0.962
		T3 VS. T1	− 1.002	1.20	0.12	0.70	0.404
		T2 VS. T1	− 1.028	1.09	0.14	0.89	0.345
	CG	T3 VS. T2	0.289	0.58	0.07	0.25	0.618
		T3 VS. T1	0.388	1.30	0.04	0.09	0.765
		T2 VS. T1	0.099	1.30	0.01	0.01	0.939
SGRQ-impact	T1	IG VS. CG	0.434	1.21	0.05	0.13	0.719
	T2	IG VS. CG	0.648	0.96	0.10	0.45	0.502
	T3	IG VS. CG	0.637	0.91	0.10	0.49	0.483
	IG	T3 VS. T2	1.270	0.48	0.38	7.04	**0.008**
		T3 VS. T1	0.505	1.20	0.06	0.18	0.675
		T2 VS. T1	− 0.765	1.05	0.10	0.53	0.467
	CG	T3 VS. T2	1.281	0.53	0.35	5.93	**0.015**
		T3 VS. T1	0.302	1.26	0.03	0.06	0.811
		T2 VS. T1	− 0.978	1.16	0.12	0.71	0.401

*IG*, intervention group; *CG*, control group; *NRS-worst*, worst breathlessness over the past 24 h assessed by the Numerical Rating Scale; *NRS-average*, average breathlessness over the past 24 h assessed by the Numerical Rating Scale; *SGRQ*, St George’s Respiratory Questionnaire; *6MWD*, 6-min walk distance; *HADS*, Hospital Anxiety and Depression Scale.

**Table 3:** Group-by-time of intervention on outcomes by adjusted GEE model

Outcomes	Time point	Intervention group		Control group		Adjusted group *time		
		Mean	SD	Mean	SD	$\beta$	95%CI	*P*
mBorg^A^	T1	4.21	1.36	4.25	1.71			
	T2	3.04	1.32	3.65	1.70	− 0.21	− 0.53, 0.10	0.175
	T3	2.79	1.51	3.10	1.62	0.06	− 0.29, 0.41	0.734
D-12-TOTAL*	T1	8.16	3.93	7.13	3.78			
	T2	5.20	3.43	5.72	3.60	− 1.27	− 2.15, − 0.39	**0.005**
	T3	4.66	3.13	5.05	3.16	− 1.41	− 2.35, − 0.46	**0.004**
D-12-physical*	T1	7.32	2.85	6.61	2.79			
	T2	5.04	2.77	5.51	3.07	− 1.02	− 1.66, − 0.37	**0.002**
	T3	4.49	2.55	4.86	2.67	− 1.13	− 1.86, − 0.40	**0.002**
D-12-affective*	T1	0.82	1.73	0.48	1.53			
	T2	0.16	0.90	0.21	0.89	− 0.27	− 0.69, 0.15	0.208
	T3	0.16	0.85	0.19	0.88	− 0.31	− 0.73, 0.12	0.160
NRS-worst^A^	T1	4.53	1.94	4.36	2.23			
	T2	3.56	1.43	3.94	1.92	− 0.28	− 0.69, 0.13	0.176
	T3	3.42	1.46	3.46	1.72	0.07	− 0.38, 0.51	0.767
NRS-average^A^	T1	2.54	1.26	2.54	1.35			
	T2	1.94	1.02	2.12	1.49	0.10	− 0.22, 0.41	0.550
	T3	1.73	1.05	1.71	1.27	0.25	− 0.08, 0.57	0.140
6MWD^B^	T1	393.04	57.45	389.82	60.90			
	T2	418.73	57.97	408.62	60.65	1.39	− 11.75, 14.53	0.836
	T3	430.22	58.30	420.35	63.92	− 1.36	− 17.95, 15.23	0.872
HADS-A^C^	T1	3.19	2.91	2.96	2.42			
	T2	2.54	2.42	2.64	2.59	0.16	− 0.55, 0.86	0.666
	T3	2.27	2.29	2.30	2.39	0.17	− 0.57, 0.9	0.662
HADS-D^C^	T1	1.92	2.59	1.74	2.51			
	T2	0.97	2.34	1.17	2.26	− 0.08	− 0.75, 0.6	0.827
	T3	0.90	2.03	0.85	1.86	0.19	− 0.51, 0.89	0.603
SGRQ-total^D^	T1	29.29	13.39	27.06	12.72			
	T2	21.50	10.51	22.88	11.53	0.28	− 1.93, 2.48	0.807
	T3	20.43	11.09	20.95	11.36	− 0.28	− 2.52, 1.95	0.804
SGRQ—symptom^D^	T1	24.94	13.41	23.00	15.37			
	T2	19.91	14.20	19.11	12.50	1.96	− 1.68, 5.59	0.291
	T3	16.61	13.79	16.75	12.00	0.32	− 3.28, 3.92	0.863
SGRQ—activity^D^	T1	46.18	16.21	43.12	15.56			
	T2	37.30	11.11	39.74	13.15	− 1.13	− 4.23, 1.98	0.477
	T3	36.07	11.66	37.05	12.01	− 1.39	− 4.53, 1.75	0.386
SGRQ—impact^D^	T1	20.85	14.61	18.98	12.58			
	T2	12.81	11.49	14.37	11.85	0.21	− 2.34, 2.76	0.869
	T3	12.55	11.82	12.97	12.48	0.20	− 2.43, 2.83	0.880

^A^Adjusted for age, BMI, smoking, exercise, HADS-total, and D-12 total scores

(* adjusted for age, BMI, smoking, exercise, HADS-total score)

^B^Adjusted for age, BMI, gender, and D-12 total scores

^C^Adjusted for age, BMI, cancer stage, surgery types, diabetes, and D-12 total score

^D^Adjusted for age, BMI, occupation, exercise, source of healthcare insurance, insomnia, mBorg score, D-12 total score, NRS-average score, NRS-worst score, and HADS total score

*NRS-worst*, worst breathlessness over the past 24 h assessed by the Numerical Rating Scale.

*NRS-average*, average breathlessness over the past 24 h assessed by the Numerical Rating Scale.

*SGRQ*, St George’s Respiratory Questionnaire.

*6MWD*, 6-min walk distance.

### Secondary outcomes

#### D-12

In the IMT group, the D-12 total score, physical subscale score, and affective subscale score at T2 and T3 were significantly lower than the scores measured at T1 (all *P* < 0.05, Table 2), suggesting improvement of breathlessness severity. In the control group, only the D-12 total score and physical score at T2 and T3 were significantly lower than the scores measured at T1 (all *P* < 0.05, Table 2). No significant between-group difference was found at each time point for D-12 total, physical, or affective aspects (all *P* > 0.05) (Table 3). The significantly greater decreases in D-12 total and D-12 physical scores were seen in the IMT group at T2 (*P*_total=_ 0.005, *P*_physical_ = 0.002) and T3 (*P*_total_ = 0.004, *P*_physical_ = 0.002) compared to the control group (Table 3).

#### NRS: worst and average breathlessness over the past 24 h

No significant group-effect and group-by-time effect of NRS-worst and NRS-average scores were observed across time (all *P* > 0.05, Tables 2 and 3). The NRS-worst score at T1 was significantly higher than the scores at T2 and T3 in the IMT group (all *P* < 0.05, Table 2). The within-group difference of NRS-worst score in the control group was significant only between T3 and T1 (*P* = 0.004, Table 2). No time-effect was observed in the NRS-average score in the IMT group, while the NRS-average score at T1 was significantly lower than T3 in the control group (*P* < 0.001, Table 2). The within-group comparisons suggested the gradually alleviated worst breathlessness severity in both groups.

#### 6MWD

The 6MWD increased in the IMT group over time, showing a significantly better exercise capacity at T2 and T3 compared with T1 (all *P* < 0.05, Table 2). Similar results were found in the control group (all *P* < 0.05, Table 2). However, there were no significant differences in 6MWD for between-group comparison (Table 2), indicating no changes in exercise capacity after IMT intervention. The group-by-time effect was not significant for the 6MWD (Table 3).

#### HADS

No significant group-effect, time-effect, and group-by-time interaction effect was observed in the scores of the HADS-A and HADS-D within the adjusted GEE module (Table 3).

#### SQRG

No significant group-effect, time-effect, and group-by-time interaction effect was observed in the scores of the SQRG-total and subscale within the adjusted GEE module (Table 3).

## Discussion

Findings of this large-scale RCT demonstrated that IMT potentially supports the improvement of breathlessness severity (mBorg and D-12) in patients with thoracic malignancies. Further identification of the sustained effect of IMT on breathlessness is necessary, mainly due to the suboptimal adherence to the intervention. Yet these findings have crucial implications in efforts to manage breathlessness among this population.

The National Institute for Health and Clinical Excellence (NICE) recommended nonpharmacological interventions as the standard care for breathlessness in patients with LC [3]. However, high-intensity interventions (e.g., intensive exercise) impose a significant burden on patients with LC and are often difficult to sustain in many cases with breathlessness [34]. IMT provides a relatively low burden on LC patients, aiming to enhance both inspiratory and expiratory muscle performance through targeted training [14, 18]. As shown by the results of this study, after IMT, there was a significant decrease in the severity of breathlessness in a short-term (8 weeks). One explanation could be that the IMT potentially improved the inspiratory muscles at a mechanical disadvantage and optimised the pattern of thoracoabdominal motion with a higher level of inspiratory resistance. Also, the improved breathing during the IMT practice can improve breathlessness as well [35]. This finding aligns with the results of a retrospective cohort study which examined the efficacy of IMT in a consistent inspiratory pressure training load alongside exercise therapy among advanced LC patients experiencing breathlessness [19].

The current study only highlights the relatively short-term effects of IMT in alleviating breathlessness, the suboptimal compliance of the participants might be one of the attributing factors. One major barrier to adherence was the inability of participants to return to the study hospital for spirometry assessments and follow-ups due to the constraints imposed by COVID-19 policies. Strategies to improve adherence were classified into four types: technical solutions (such as decreased frequency and intensity); educational programs; behavioral interventions; and social support interventions [36]. The updated IMT protocol used in this study has been divided into two sessions and the intensity has been built up slowly according to participants’ tolerance. Future studies can include some behavioral change components to improve participants’ adherence further, for example, more effective reminders, regular monitoring, or reward-based initiation. The protocol could be modified to provide in-person home visits as an option, which could help with subsequent tracking effectively. Also, integrating some social platforms, such as the WeChat App, into self-management intervention could be another promising strategy for improving adherence. Additionally, the between-group differences in D-12 total score and BMI at baseline in this study indicated the benefits of using stratified randomisation in future studies to minimize statistical bias.

This study found no significant group-time interaction effects on the QoL, consistent with two systematic reviews showing that the effects of IMT on QoL among LC patients remain ambiguous [14, 18]. QoL of patients with thoracic malignancies may be impacted by multiple factors (e.g., the severity and the number of cancer-related symptoms, detreated lung function etc.), which may reflect differently in a subjective measure of the QoL [37]. The findings of this study on anxiety and depression were the same as previous studies, indicating that breathing exercise programs (including IMT and incentive deep-breathing exercise) could not significantly improve the emotional status of patients with LC [34]. However, an undeniable fact is that high levels of anxiety and depression in patients with LC are primarily attributed to progressive disease and worsening symptoms in LC patients [38, 39]. Also, anxiety and depression were regarded as factors associated with dropout and non-adherence with IMT in previous literature [40]. Thus, a combination of psychological support (e.g., cognitive-behavioral therapy) with the IMT intervention could be considered in future studies to promote participants’ engagement and maximise the interventional effects among this population.

In the pilot study, spirometry was used to assess lung function but was excluded from the current trial due to two reasons: (a) the pilot study indicated no change in lung function at any assessments, suggesting minimal expected improvements in lung function among this population with advanced cancer. Thus, the preservation of stable breathlessness emerges as a critical concern. (b) Current literature consistently shows a lack of association between lung function and perceived breathlessness improvement [41]. Given this insight, the current study included the 6MWT based on the expert’s recommendations. A meta-analysis showed similar findings, indicating that breath exercise (including IMT intervention) did not improve the 6MWD in LC patients undergoing surgery [42]. A retrospective pilot study has reported conflicting findings, suggesting that IMT combined with pulmonary rehabilitation appears effective on exercise capacity in non-small cell LC patients receiving radiotherapy [43]. Additionally, a recent RCT observed a 6-week IMT combined with aerobic exercise led to improved exercise capacity (measured by 6MWT) in LC patients after surgery [13]. These results could potentially be attributed to the sub-optimal adherence to the IMT intervention and the heterogeneity of subsequent self-assessment of 6MWD using a phone at home. To overcome this challenge, it would be advisable for future studies to encourage patients to visit the hospital for a standardized measurement. Alternatively, employing healthcare practitioners or trained researchers for home or community-based evaluations could enhance the accuracy of assessments.

## Strengths and limitations

To our knowledge, this study represents the first large-scale RCT to thoroughly investigate the effects of IMT among patients with thoracic malignancies. This study focuses on the patients’ self-practice at home, indicating a practical combination of the intervention into their daily care routines. The adherence to IMT practice was reported by diary alone across the COVID-19 outbreak, limiting interpretation of results. The incorporation of electronic monitoring has been suggested to assess adherence and is considered the ‘gold standard’ for measuring adherence in similar interventions [44]. Due to resource constraints, the blinding of participants was not achieved. Although the 6MWT is simple to self-administer with standardized instructions provided, having this test at home without direct supervision during the COVID-19 outbreak may affect the consistency and generalizability of the results. Also, given that the 6MWD tends to increase after 3 walks due to a learning effect [45], 1 to 2 practice tests are suggested before determining an individual’s exercise capacity in future studies. Future studies should consider adopting a sham-controlled design, such as instructing participants to use the same device with no or minimal resistance, to further strengthen the internal validity and minimise potential bias.

## Conclusions

The IMT intervention potentially reduced breathlessness in patients with thoracic malignancies in the short term. Further study could incorporate a follow-up and advanced monitoring strategy to better explore its long-term effects on breathlessness in patients with thoracic malignancies.

## Data Availability

No datasets were generated or analysed during the current study.

## References

[1] Leiter A, Veluswamy RR, Wisnivesky JP (2023) The global burden of lung cancer: current status and future trends. Nat Rev Clin Oncol 20(9):624–639. 10.1038/s41571-023-00798-3 37479810 10.1038/s41571-023-00798-3

[2] World Health Organization (2023) Lung Cancer. https://www.who.int/news-room/fact-sheets/detail/lung-cancer . Accessed 11 Apr 2024

[3] National Institute for Health and Care Excellence (2023) Lung cancer: diagnosis and management. www.nice.org.uk/guidance/ng122. Accessed Jan 2024 31211540

[4] Nomori H, Horio H, Fuyuno G, Kobayashi R, Yashima H (1996) Respiratory muscle strength after lung resection with special reference to age and procedures of thoracotomy. Eur J Cardiothorac Surg 10:352–358 8737692 10.1016/s1010-7940(96)80094-7

[5] Shin J, Kober K, Wong ML et al (2023) Systematic review of the literature on the occurrence and characteristics of dyspnea in oncology patients. Crit Rev Oncol Hematol 181:103870. 10.1016/j.critrevonc.2022.103870 36375635 10.1016/j.critrevonc.2022.103870 PMC12364023

[6] Lo SB, Ruprecht AL, Post KE et al (2024) Dyspnea-related dimensions and self-efficacy: Associations with well-being in advanced lung cancer. J Pain Symptom Manage 67(5):366–374. 10.1016/j.jpainsymman.2024.01.032 10.1016/j.jpainsymman.2024.01.032 PMC11032235 38307373

[7] Dy SM, Lorenz KA, Naeim A et al (2008) Evidence-based recommendations for cancer fatigue, anorexia, depression, and dyspnea. J Clin Oncol 26(23):3886–3895. 10.1200/JCO.2007.15.9525 10.1200/JCO.2007.15.9525 18688057

[8] Patel MI, Williams DC, Wohlforth C et al (2015) Are patients with thoracic malignancies at risk for uncontrolled symptoms? J Oncol Pract 11(1):e98–e102. 10.1200/JOP.2014.001502 25271246 10.1200/JOP.2014.001502

[9] Uronis H, Currow D, McCrory D et al (2008) Oxygen for relief of dyspnoea in mildly- or non-hypoxaemic patients with cancer: a systematic review and meta-analysis. Br J Cancer 98:294–299. 10.1038/sj.bjc.6604161 18182991 10.1038/sj.bjc.6604161 PMC2361446

[10] Geddes E, Reid WD, Brooks D et al (2010) A primer on inspiratory muscle trainers. Available at: http://dev.ersnet.org/uploads/Document/WEB_CHEMIN_419_1163420850.pdf. Accessed 13 Jan 2025

[11] Ammous O, Feki W, Lotfi T et al (2023) Inspiratory muscle training, with or without concomitant pulmonary rehabilitation, for chronic obstructive pulmonary disease (COPD). Cochrane Database of Systematic Reviews 1(1):CD013778. 10.1002/14651858.CD013778.pub2 36606682 10.1002/14651858.CD013778.pub2 PMC9817429

[12] Zhang F, Zhong Y, Qin Z et al (2021) Effect of muscle training on dyspnea in patients with chronic obstructive pulmonary disease: a meta-analysis of randomized controlled trials. Medicine (Baltimore) 100(9):e24930. 10.1097/MD.0000000000024930 33655957 10.1097/MD.0000000000024930 PMC7939163

[13] Liu JF, Kuo NY, Fang TP et al (2021) A six-week inspiratory muscle training and aerobic exercise improves respiratory muscle strength and exercise capacity in lung cancer patients after video-assisted thoracoscopic surgery: a randomized controlled trial. Clin Rehabil 35(6):840–850. 10.1177/0269215520980138 33307766 10.1177/0269215520980138

[14] Nguyen NM, Latiers F, Nana FA (2023) The effect of inspiratory muscle training in patients with lung cancer after surgery: a systematic review. Rehabil Oncol 41(4):202–212. 10.1097/01.REO.0000000000000352

[15] McConnell AK. (2013) Respiratory muscle training theory and practice. UK: Elsevier Health Sciences, pp 403–405

[16] Molassiotis A, Charalambous A, Taylor P et al (2015) The effect of resistance inspiratory muscle training in the management of breathlessness in patients with thoracic malignancies: a feasibility randomised trial. Support Care Cancer 23(6):1637–1645. 10.1007/s00520-014-2511-x 25417042 10.1007/s00520-014-2511-x

[17] Rao JB, Bansal K (2024) Breathing exercises in lung cancer-a systematic review. Pakistan Heart Journal 57(1):388–398

[18] Tórtola-Navarro A, Gallardo-Gómez D, Álvarez-Barbosa F et al (2024) Cancer survivor inspiratory muscle training: systematic review and Bayesian meta-analysis. BMJ Support Palliat Care 13(e3):e561–e569. 10.1136/spcare-2022-003861 36216456 10.1136/spcare-2022-003861

[19] Sakai Y, Yamaga T, Yamamoto S et al (2023) Effects and usefulness of inspiratory muscle training load in patients with advanced lung cancer with dyspnea. J Clin Med 12(10):3396. 10.3390/jcm12103396 37240502 10.3390/jcm12103396 PMC10219417

[20] Paiva DN, Assmann LB, Bordin DF et al (2015) Inspiratory muscle training with threshold or incentive spirometry: which is the most effective? Revista Portuguesa de Pneumologia (English Edition) 21(2):76–81. 10.1016/j.rppnen.2014.05.005 10.1016/j.rppnen.2014.05.005 25926370

[21] Oxberry SG, Bland JM, Clark AL et al (2012) Minimally clinically important difference in chronic breathlessness: every little helps. Am Heart J 164(2):229–235. 10.1016/j.ahj.2012.05.003 22877809 10.1016/j.ahj.2012.05.003

[22] Yorke J, Swigris J, Russell AM et al (2011) Dyspnea-12 is a valid and reliable measure of breathlessness in patients with interstitial lung disease. Chest 139(1):159–164. 10.1378/chest.10-0693 20595454 10.1378/chest.10-0693 PMC3035488

[23] Liu XL, Peng B, Wang T et al (2025) Psychometric validation of the simplified Chinese version of the dyspnoea-12 questionnaire for patients with primary lung cancer. Healthcare 13(2):201. 10.3390/healthcare13020201 39857227 10.3390/healthcare13020201 PMC11764697

[24] Gift AG, Narsavage G (1998) Validity of the numeric rating scale as a measure of dyspnea. Am J Crit Care 7(3):200–204 9579246

[25] Pires IM, Denysyuk HV, Villasana MV et al (2022) Development technologies for the monitoring of six-minute walk test: a systematic review. Sensors 22(2):581. 10.3390/s22020581 35062542 10.3390/s22020581 PMC8782011

[26] Giannitsi S, Bougiakli M, Bechlioulis A et al (2019) 6-minute walking test: a useful tool in the management of heart failure patients. Ther Adv Cardiovasc Dis 13:1753944719870084. 10.1177/1753944719870084 31441375 10.1177/1753944719870084 PMC6710700

[27] Rashidmiya QM (2012) Assessment of health related quality of life in lung cancer patients: validity of St. George Respiratory Questionnaire. Master's thesis, Rajiv Gandhi University of Health

[28] Lima LN, da Silva RA, Gross JL et al (2009) Assessment of pulmonary function and quality of life in patients submitted to pulmonary resection for cancer. J Bras Pneumol 35(6):521–528. 10.1590/s1806-37132009000600005 19618032 10.1590/s1806-37132009000600005

[29] Chan-Yeung MM, Ooi GC et al (2002) Validation of the Hong Kong Chinese version of the St. George Respiratory Questionnaire in patients with bronchiectasis. Chest 122(6):2030–2037. 10.1378/chest.122.6.2030 12475843 10.1378/chest.122.6.2030

[30] Li Q, Lin Y, Hu C et al (2016) The Chinese version of hospital anxiety and depression scale: psychometric properties in Chinese cancer patients and their family caregivers. Eur J Oncol Nurs 25:16–23. 10.1016/j.ejon.2016.09.004 27865248 10.1016/j.ejon.2016.09.004

[31] de Mol M, Visser S, Aerts J et al (2020) The association of depressive symptoms, personality traits, and sociodemographic factors with health-related quality of life and quality of life in patients with advanced-stage lung cancer: an observational multi-center cohort study. BMC Cancer 20:1–14 10.1186/s12885-020-06823-3 PMC7236491 32423432

[32] Dudgeon DJ, Kristjanson L, Sloan JA et al (2001) Dyspnea in cancer patients: prevalence and associated factors. J Pain Symptom Manage 21(2):95–102 11226761 10.1016/s0885-3924(00)00258-x

[33] Ballinger GA (2004) Using generalized estimating equations for longitudinal data analysis. Organ Res Methods 7(2):127–150. 10.1177/1094428104263672

[34] Ma RC, Zhao Y, Liu X et al (2021) Multimodal exercise program: a pilot randomized trial for patients with lung cancer receiving surgical treatment. Clin J Oncol Nurs 25(3):E26–E34. 10.1188/21.CJON.E26-E34 34019026 10.1188/21.CJON.E26-E34

[35] Barton R, English A, Nabb S et al (2010) A randomised trial of high vs low intensity training in breathing techniques for breathless patients with malignant lung disease: a feasibility study. Lung Cancer 70(3):313–319 20392515 10.1016/j.lungcan.2010.03.007

[36] Bender B, Milgrom H, Apter A (2003) Adherence intervention research: what have we learned and what do we do next? J Allergy Clin Immunol 112(3):489–494 13679805 10.1016/s0091-6749(03)01718-4

[37] Polanski J, Jankowska-Polanska B, Rosinczuk J et al (2016) Quality of life of patients with lung cancer. OncoTargets and Therapy 9:1023–1028. 10.2147/OTT.S100685 27013895 10.2147/OTT.S100685 PMC4778772

[38] Jung JY, Lee JM, Kim MS et al (2018) Comparison of fatigue, depression, and anxiety as factors affecting posttreatment health-related quality of life in lung cancer survivors. Psychooncology 27(2):465–470. 10.1002/pon.4513 28755492 10.1002/pon.4513

[39] Wang X, Ma X, Yang M et al (2022) Proportion and related factors of depression and anxiety for inpatients with lung cancer in China: a hospital-based cross-sectional study. Support Care Cancer 30(6):5539–5549. 10.1007/s00520-022-06961-3 10.1007/s00520-022-06961-3 PMC9046329 35318530

[40] Keating A, Lee A, Holland AE (2011) What prevents people with chronic obstructive pulmonary disease from attending pulmonary rehabilitation? A systematic review. Chron Respir Dis 8(2):89–99. 10.1177/1479972310393756 21596892 10.1177/1479972310393756

[41] Vestbo J, Hurd SS, Agustí AG et al (2013) Global strategy for the diagnosis, management, and prevention of chronic obstructive pulmonary disease: GOLD executive summary. Am J Respir Crit Care Med 187(4): 347–365. 10.1164/rccm.201204-0596PP 10.1164/rccm.201204-0596PP 22878278

[42] Wang YQ, Liu X, Jia Y et al (2019) Impact of breathing exercises in subjects with lung cancer undergoing surgical resection: a systematic review and meta-analysis. J Clin Nurs 28(5–6):717–732. 10.1111/jocn.14696 30357997 10.1111/jocn.14696

[43] Do J, Lee SH, Kim SA et al (2023) The effects of inspiratory muscle training with pulmonary rehabilitation on NSCLC patients during radiation therapy: a pilot clinical study. Thorac Cancer 14(17):1567–1573. 10.1111/1759-7714.14899 37078293 10.1111/1759-7714.14899 PMC10260495

[44] George J, Kong DC, Stewart K (2007) Adherence to disease management programs in patients with COPD. Int J Chron Obstruct Pulmon Dis 2(3):253–262 18229563 PMC2695203

[45] Wu G, Sanderson B, Bittner V (2003) The 6-minute walk test: how important is the learning effect? Am Heart J 146(1):129–133. 10.1016/S0002-8703(03)00119-4 12851620 10.1016/S0002-8703(03)00119-4

